# A randomized 3-month, parallel-group, controlled trial of CALMA m-health app as an adjunct to therapy to reduce suicidal and non-suicidal self-injurious behaviors in adolescents: study protocol

**DOI:** 10.3389/fpsyt.2023.1087097

**Published:** 2023-07-20

**Authors:** Demián Emanuel Rodante, Luciana Carla Chiapella, Ramiro Olivera Fedi, Eliana Belén Papávero, Kim L. Lavoie, Federico Manuel Daray

**Affiliations:** ^1^Facultad de Medicina, Universidad de Buenos Aires, Instituto de Farmacología, Buenos Aires, Argentina; ^2^“Dr. Braulio A. Moyano” Neuropsychiatric Hospital, Buenos Aires, Argentina; ^3^FORO Foundation for Mental Health, Buenos Aires, Argentina; ^4^Pharmacology Area, Faculty of Biochemical and Pharmaceutical Sciences, National University of Rosario, Rosario, Argentina; ^5^Consejo Nacional de Investigaciones Científicas y Técnicas, Buenos Aires, Argentina; ^6^Pedro de Elizalde Children’s General Hospital, Buenos Aires, Argentina; ^7^Montreal Behavioural Medicine Centre, Centre Intégré Universitaire de Santé et de Services Sociaux du Nord-de-l’Île-de-Montréal, Montreal, QC, Canada; ^8^Department of Psychology, Université du Québec à Montréal, Montreal, QC, Canada

**Keywords:** dialectical behavioral therapy, mobile health application, suicide prevention, suicide and self-harm, child and adolescent psychiatry

## Abstract

**Background:**

Suicidal and non-suicidal self-injurious behaviors are among the leading causes of death and injury in adolescents and youth worldwide. Mobile app development could help people at risk and provide resources to deliver evidence-based interventions. There is no specific application for adolescents and young people available in Spanish. Our group developed CALMA, the first interactive mobile application with the user in Spanish, which provides tools based on Dialectical Behavioral Therapy to manage a crisis of suicidal or non-suicidal self-directed violence with the aim of preventing suicide in adolescents and youth.

**Methods:**

To test the effectiveness, safety and level of engagement of the CALMA app in people aged 10 to 19 who are treated in mental health services of two public hospitals, we will conduct a parallel-group, two-arm randomized controlled trial. Participants will be assessed face-to-face and *via* video call at four timepoints: day-0 (baseline), day-30, day-60, and day-90. A total of 29 participants per group will be included. Change in the frequency of suicidal and non-suicidal self-injurious behaviors will be compared between groups, as well as the level of emotional dysregulation, level of app engagement and time of psychiatric admission during the follow-up period.

**Discussion:**

This study is particularly relevant to young people given their widespread use of mobile technology, while there are currently no available smartphone app-based self-guided psychological strategies in Spanish that attempt to reduce suicidal behavior in adolescents who are assisted in the public health sector from low and middle-income countries in Latin America.

**Clinical trial registration:**

https://clinicaltrials.gov/, NCT05453370.

## Introduction

Suicidal behaviors are among the leading causes of death and injury worldwide. Over 700,000 people die by suicide every year (1). For every suicide, more than 30 people attempt suicide (2), and the lifetime prevalence of suicide attempts (SA) among the general population is approximately 2.7% (3). The magnitude of the problem is even higher if we consider the prevalence of suicidal ideation (SI), being estimated at 8.3% in the Southern Cone of Latin America (4). Altogether, these data suggest that the impact of suicidal thoughts and behavior is a significant public health problem around the world (5). Regarding Non suicidal self-injury (NSSI) rates are also high, particularly among adolescents (6), with approximately 20% of high school students reporting at least one episode of NSSI in the past 12 months (7). This is in particular importance, as NSSI is a significant risk factor for suicidal behaviors (8, 9).

The suicide mortality rate Argentina is 7.47 per hundred thousand inhabitants (10). Lately, there was a gradual and sustained growth in the suicide rate in individuals between 15 and 24 years of age (10). In 2019, of the total of 3,297 deaths by suicide, 868 deaths occurred in those between 15 and 24 years of age, representing the second cause of death (after traffic accidents) (10). In adolescents (people between 10 and 19 years old according to the WHO) a total of 459 deaths by suicide occurred in that year (10). This epidemiological transition observed in Argentina coincides with what has been reported internationally and confronts us with a new challenge.

The evidence on the efficacy and effectiveness of suicide prevention strategies, such as Dialectical Behavioral Therapy (DBT), have been described (11–14). Indeed, DBT prioritizes suicidal behavior as the primary goal of treatment. In a meta-analysis of 18 RCTs, DeCou et al. demonstrated a significant pooled effect for DBT compared to control groups for self-directed violence (suicidal and NSSI behavior; *d* = −0.324, 95% CI = −0.471 to −0.176); however, there was no significant pooled effect of DBT for suicidal ideation (15). A more recent review, which evaluated the benefits and harms of psychological therapies for borderline personality disorder, identified DBT as having the largest number of primary trials, finding it to be the most effective psychotherapy at reducing self-harm (SMD-0.28, 95% CI-0.48 to-0.7; 7 trials, 376 participants) compared to TAU (16). Furthermore, in adolescents, 19 weeks of DBT was associated with a significantly lower frequency of self-harm episodes and suicidal ideation at post-treatment, 1 year, and 3 years follow-up (17, 18). In addition, some studies with similar results have added data regarding the feasibility of implementing DBT in adolescents, even at a community level, a necessary hallmark of the program (19, 20). Recently, a meta-analysis by Witt et al. concluded that there was a lower recurrence rate of self-harm (intentional self-harm) for adolescent DBT (30%) compared with treatment as usual, enhanced usual care, or alternative psychotherapy (43%) in repeated post-intervention self-harm in four trials (OR 0.46, 95% CI 0.26 to 0.82; *N* = 270; *k* = 4; high-certainty evidence) (21).

Despite this, and due to the short time to intervene in the suicidal process (22, 23), a crucial aspect of their effectiveness in daily practice is accessibility. This is why, beyond employing effective interventions to prevent suicide, they must be quickly available at the time of crisis.

### Rationale

There has been rapid growth in the development and use of digital intervention technologies (24). New platforms to provide evidence-based interventions, universally, economically, and quickly are needed (25, 26). Smartphones appear to be a good alternative considering the high penetration of these devices locally (26), and the development of mobile applications (apps) to prevent suicidal behavior might could support individuals at risk (27, 28) and provide resources to deliver evidence-based interventions (29). Several developments have occurred, and protocols for effectiveness evaluation have also been published (30–40), but few included descriptive reports on the development process and feasibility (36, 41–43).

Since the app review by Larsen in 2016 (44), nearly twice as many apps designed to reduce suicide have been introduced in app stores (45). Most of the apps available provide information about suicidal behavior/NSSI or general information about mental health. In general, they include functions that allow interaction with the user to help manage the crisis, typically using two types of suicide prevention strategies: (a) providing emergency resources to the user (non-existent or inaccurate suicide crisis helpline phone numbers in a few of them (46); (b) providing management/reduction of (acute) general or suicidal/NSSI urges and behavior, with a focus on developing a safety plan or using some type of coping strategy (45–48). Unfortunately, several mobile apps targeting suicidal behavior have not employed evidence-based treatment approaches (44, 49). Furthermore, to our knowledge, no specific app for adolescents and young people is available in Spanish (only one app, Prevensuic, is in Spanish, but it is not interactive, it does not present tools or coping skills, and it is not specifically targeting adolescent) (35, 50). Our group developed CALMA (the Spanish word for “calm”), the first user-interactive mobile app in Spanish. CALMA provides evidence-based tools to manage a suicidal or non-suicidal self-directed violence crisis with the goal of preventing death by suicide (51). CALMA also interacts with the user between crises by promoting activities that reduce their vulnerability to future crises, and providing psychoeducation about suicide and its prevention (51).

Our research group carried out a pilot cluster randomized controlled trial with 4 weeks of follow-up that provided initial evidence on the safety and acceptability of the app for reducing self-injurious thoughts and behaviors when used as an adjunct to conventional DBT therapy (52). Being the first pilot study, this was carried out in a group of patients already under a DBT treatment program in a private clinical setting. Six DBT Skills Training groups were randomized to the intervention (DBT + CALMA) or control (DBT alone). Although efficacy was not the main outcome, since it was a pilot study, the results showed a high probability of decreased suicidal ideation, suicidal plan, suicidal gesture (or threat), thoughts about Non-Suicidal Self-Injury (NSSI), and NSSI in the group receiving CALMA app. Moreover, CALMA showed good acceptability as an adjunct to therapy targeting suicidal and non-suicidal self-injury behaviors measured with the User Experience Questionnaire in its short version (UEQ-s) (52).

Since the pilot study was not powered for efficacy, and based on these encouraging initial findings, in this project we propose to scale the intervention to a larger group of patients, focus it on adolescents, and include public hospitals not specialized in DBT.

## Objectives

### General objective

To evaluate the efficacy, safety and acceptability of CALMA, a suicide prevention app for smartphones, to reduce the frequency of self-injurious thoughts and behaviors in adolescents who are assisted in a Mental Health service at two Public hospitals.

### Specific objectives

#### Primary specific objectives

- Compare the change in the frequency of self-injurious behaviors in the participants who receive the app with the frequency in those who do not receive the application.

#### Secondary specific objectives

- Analyze the frequency of self-injurious thoughts in the participants who receive the application with the frequency in those who do not receive the app.
- Establish that there is no increase in the frequency of self-injurious thoughts and behaviors in those participants who receive the application compared to the baseline levels at study onset.
- To explore whether the effectiveness of CALMA in reducing the frequency of self-injurious thoughts and behaviors is mediated by improved emotional regulation.
- Describe the levels of app engagement by the adolescent participants who used it during this study.
- To compare the time to psychiatric admission during the follow-up period in the participants enrolled who received the app with those participants who did not receive the app.

## Methods and analysis

### Trial design

This protocol describes the methodology of a parallel-group, two-arm randomized controlled trial, which will employ an intervention condition (CALMA app) and a control condition (Treatment as usual-TAU) with a 3-month follow-up for each participant. Participants will be assessed at four timepoints: day-0 (baseline), day-30, day-60, and day-90.

The intervention group will receive the CALMA app and continue with the usual treatment in a public hospital mental health service. The CALMA app will be downloaded with the assistance of a team member into the participant’s smartphone during the first interview. In each follow-up interview (30-days and 60-days), the use of the app will be reinforced. The control group (TAU) will not receive the app but will keep doing the usual treatment provided in public hospitals throughout the study. The usual treatment in public hospitals in Argentina consists of a weekly visit to a psychologist and a monthly visit to a psychiatrist (in case the patient receives any pharmacological treatment). In the public hospitals of Argentina, there are no DBT programs, and most of the interventions are psychoanalytic.

### Study setting

Young people aged 10 to 19 years (WHO definition of adolescence) who attend a Mental Health Services of the Pedro de Elizalde Children’s General Hospital (in the City of Buenos Aires), and the Children’s Hospital “Sor María Ludovica” from the city of La Plata (Province of Buenos Aires) who have presented some type of self-injurious behavior (with or without suicidal intent) the month before the evaluation will be randomly assigned to one of the arms of study.

### Patient eligibility

#### Subjects

Patients aged 10 to 19 who attend on as outpatient basis or have been hospitalized in Mental Health Services and who have a smartphone where the application can be installed and used are eligible to participate in the study. In addition, participants must have had suicidal behavior in the 4 weeks before entering the study. The SITBI assessment questions will be used to define suicidal behavior: suicide attempt [“made a suicide attempt (i.e., purposefully hurt yourself with at least some intent to die)?”], exhibited a suicidal gesture (Have you ever done something to lead someone to believe that you wanted to kill yourself when you really had no intention of doing so?) or self-injurious behavior (Have you ever done something with the purpose of hurting yourself without wanting to die?–for example, cutting or burning-) in the last month before entering the study (53, 54).

The participant must have plans to continue treatment in the next 3 months, have the ability to provide assent/consent for cognitive or language reasons, and the functional capacity to use the application, which will be operationally determined by a score above 30 on the Self-Care motor domain, above 10 on the Communication cognitive domain and above 14 on the Cognitive domain of Social Knowledge in the Functional Independence Measurement scale (46).

### Intervention

#### Implementation of the intervention

**Figure 1:**
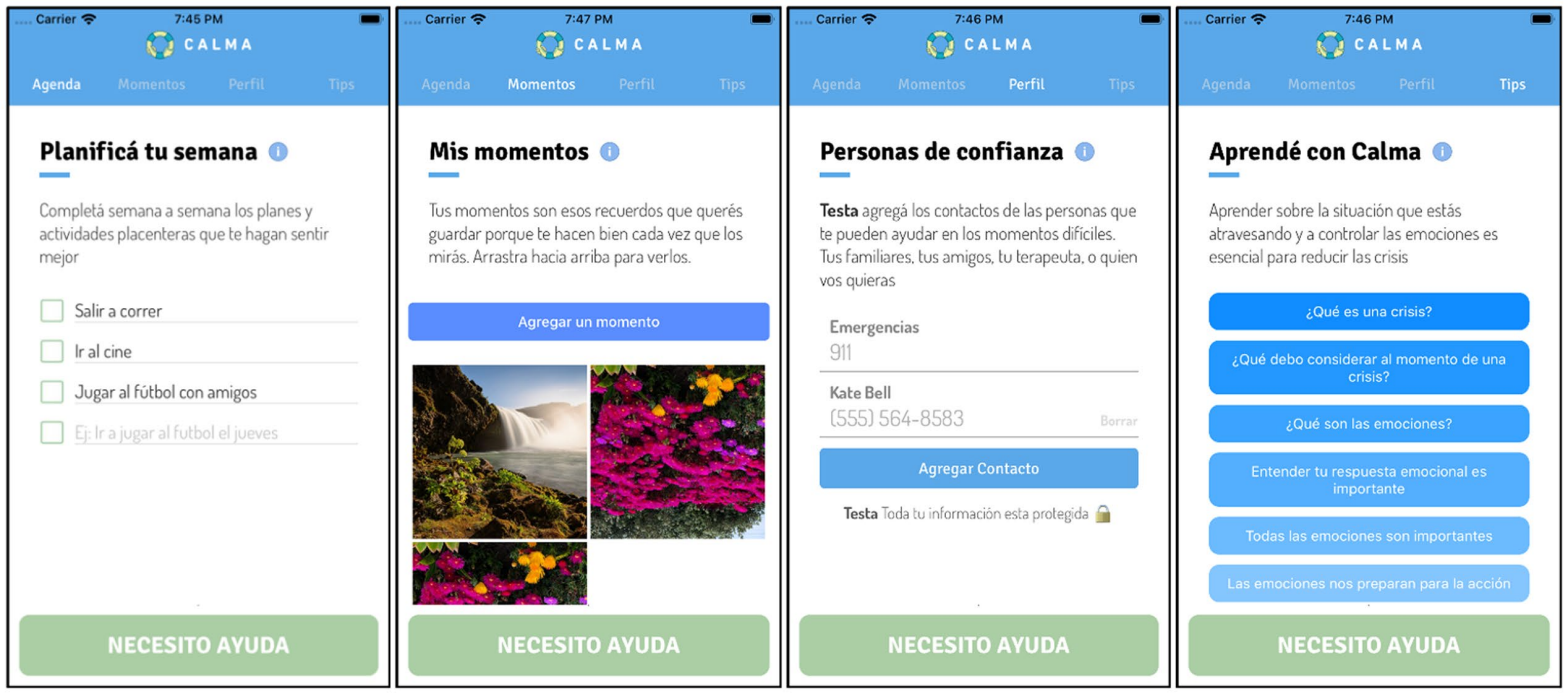
Functions of “Out of Crisis” mode of CALMA: Moments, Agenda, Profile and Tips.

CALMA is an app for smartphones that interacts with the user, providing evidence-based tools to prevent suicide; it is openly available and free to download on Apple or Google market place. It was developed, with contribution of mental health professionals, by our research group which are also mental health professionals. CALMA is based on the DBT program, which is one of the third-wave cognitive-behavioral therapies and seeks to train consultants with self-injurious behavior with and without suicidal intent in emotional regulation skills in order to manage their emotions more effectively (13, 55). CALMA integrates DBT strategies in two modalities: “Out of crisis” in which the user has some vulnerability reduction strategies to reduce the probability of crisis appearance, and the “I Need Help” modality (aimed at helping the user in crisis) constituted first with problem-solving strategies (DBT always gives the opportunity to solve the problem that triggered the crisis) and then, if the intensity of the crisis does not decrease, it implements distress tolerance or emotional regulation DBT skills dependent on the level of distress rated by the user (concept derived from the *breaking point* of DBT skills). A detailed description of the development of CALMA has been described elsewhere (51). All the features of CALMA app are based on face-to-face interventions that have shown efficacy in the reduction of self-injurious behavior and that are part of the central axis of the DBT program such as problem-solving (54), emotion regulation (13, 55, 56), distress tolerance (13, 55, 56) and also access to emergency contacts at the time of the crisis (31, 35). The app offers these interventions sequentially, and users can select which to employ. To guarantee the fidelity of the CALMA app to the model, DBT therapists have been included as stakeholders in the design of the intervention. Also, participants’ opinion was considered (52). In the management of an emotional crisis, the CALMA app follows the steps of crisis management and telephone coaching, initially going through the problem-solving strategy until reaching discomfort tolerance or emotional regulation skills as self-management strategies (11, 57, 58). The “Out of Crisis” modality consists of four functions that are identified in the initial screen of the app (Figure 1). The “Moments” function is based on the Inventory of Reasons to Live (59), considering that reasons for living reduce the risk of suicide (59, 60). In “Moments,” the user can add photos, videos, audios, songs, phrases, or anything that generates well-being and works as an anchor to life. The (b) “Agenda” function is based on evidence that positive events, such as pleasurable activities, serves as a protective factor for SI, thus reducing vulnerability to future suicidal crises (61). Moreover, engagement in positive events serves as a buffer between stressful adverse events and SI (62). The (c) “Profile” function provides the option for the user to add names and telephone numbers of people who can be contacted during a crisis if the crisis is not able to be resolved with the “Emergency Card” (see later). The (d) “Tips” function provides psychoeducational material related to suicidal crises and how to prevent unwanted behaviors during these crises. The user receives a weekly notification as a reminder of these sections. The “I need help” modality consists of interventions based on DBT skills. The skills were designed to be easily accessed during a crisis by the user activating the “I need help” button at the bottom of the home screen. All interventions are presented in a card format.

**Figure 2:**
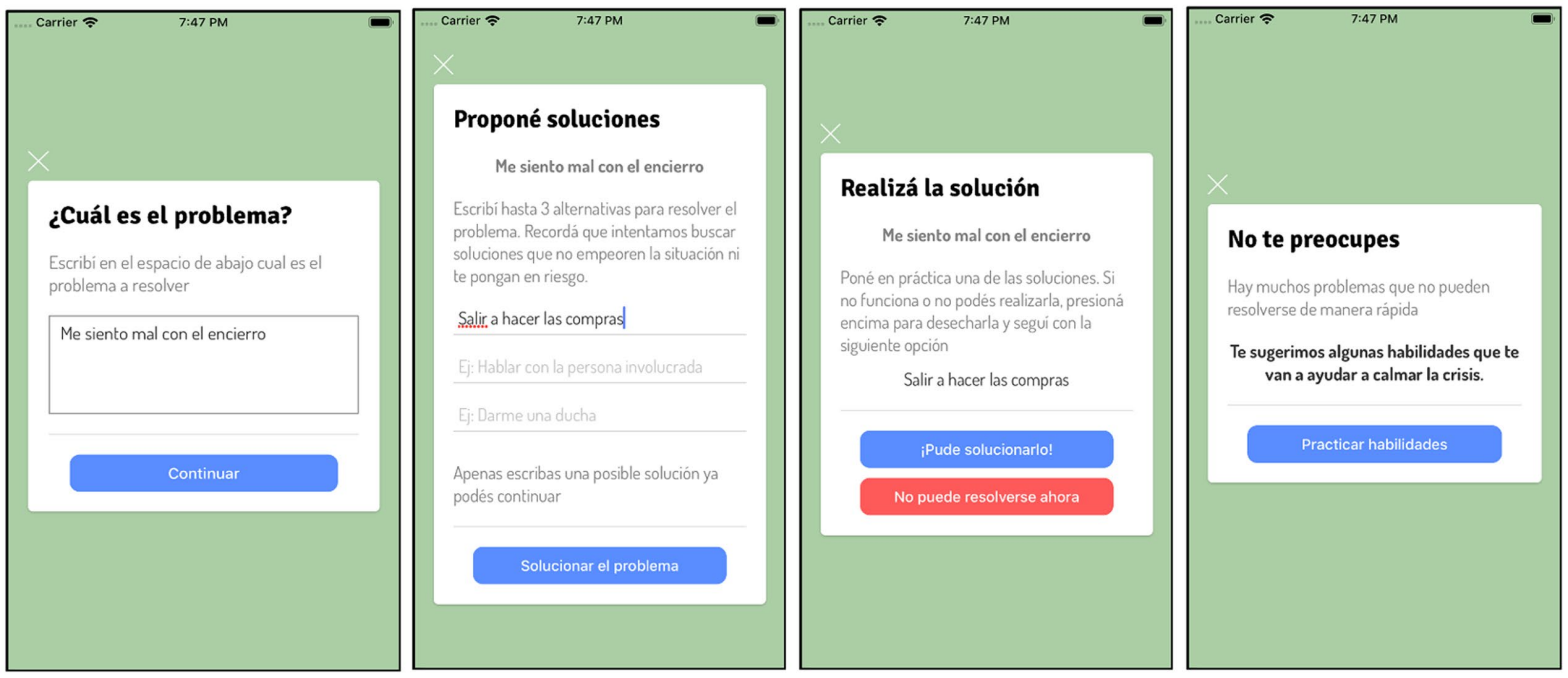
“I need help” mode of CALMA: Problem-solving Card.

**Figure 3:**
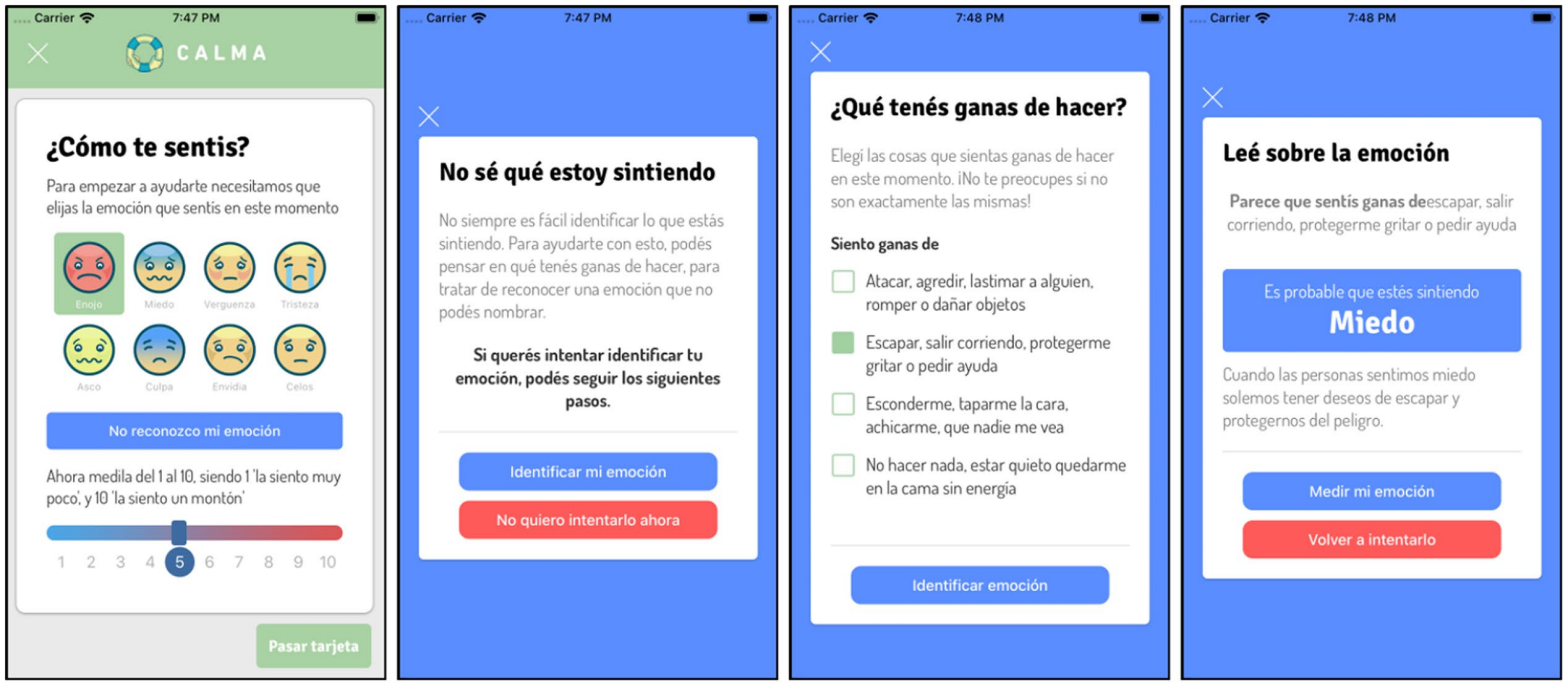
“I need help” mode: CALMA thermometer.

The “Problem-solving Card” is the first card presented and helps the user evaluate if the problem that triggered the crisis can be addressed using problem-solving (Figure 2), which is an effective strategy for people experiencing SI and SA (63–66). If the problem cannot be addressed using problem solving, the next step is to use the “CALMA thermometer” and “DBT Skills Cards.” The “CALMA thermometer” allows the user to identify what emotion he or she is feeling and to quantify the intensity of the emotion using a 10-point thermometer (Figure 3).

**Figure 4:**
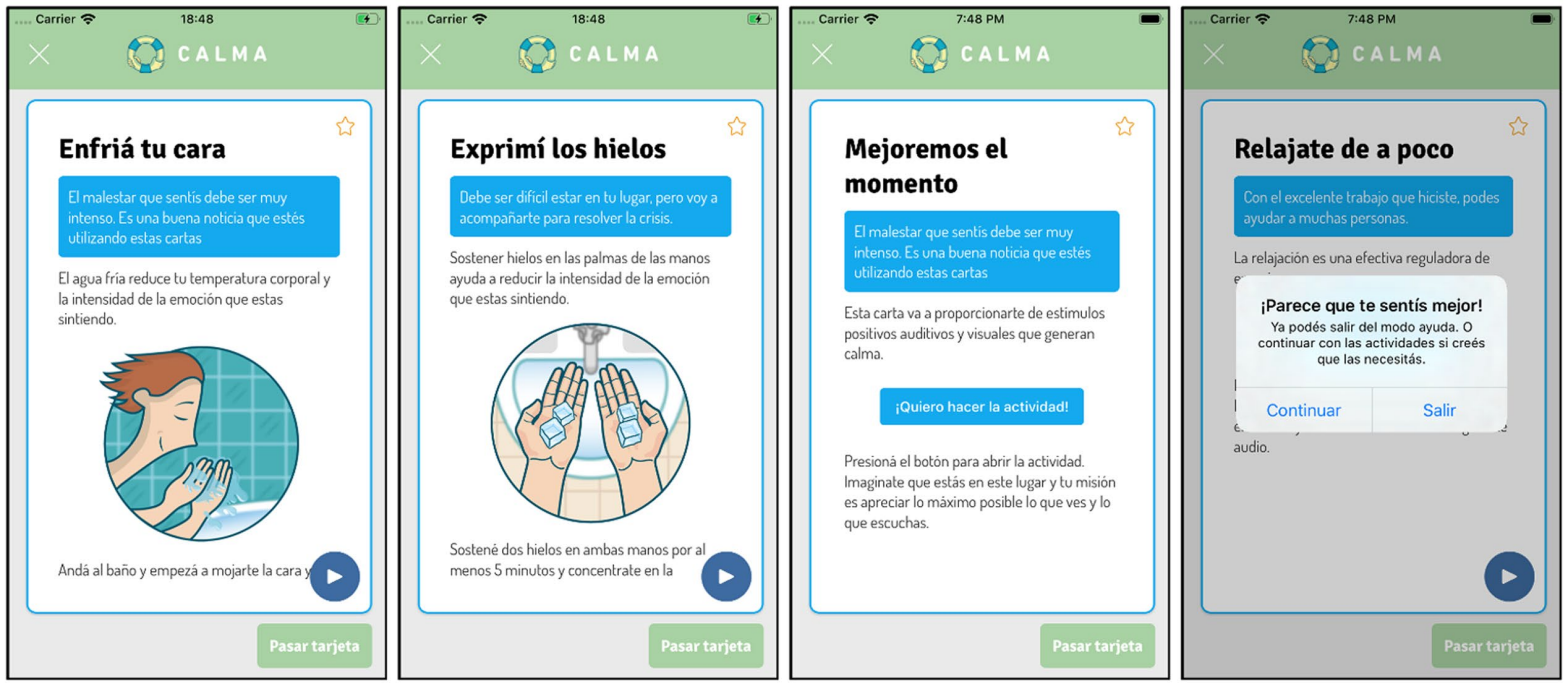
“I need help” mode of CALMA: DBT Skills Cards.

“DBT Skill Cards”: CALMA includes different cards with DBT skills (interventions) to manage emotional crises (Figure 4). Each card provides a specific DBT skill and the instruction on how to do it. The user can read, view, or listen to the content of each card and choose to perform the skill or discard it. The DBT Skill Cards are based fundamentally in two groups of DBT skills, emotional regulation and distress tolerance. Once the skill is applied, the user must re-score the intensity of the emotion via the CALMA thermometer. According to the effectiveness of the intervention, the app follows an algorithm designed to select the next card that the user will receive.

**Figure 5:**
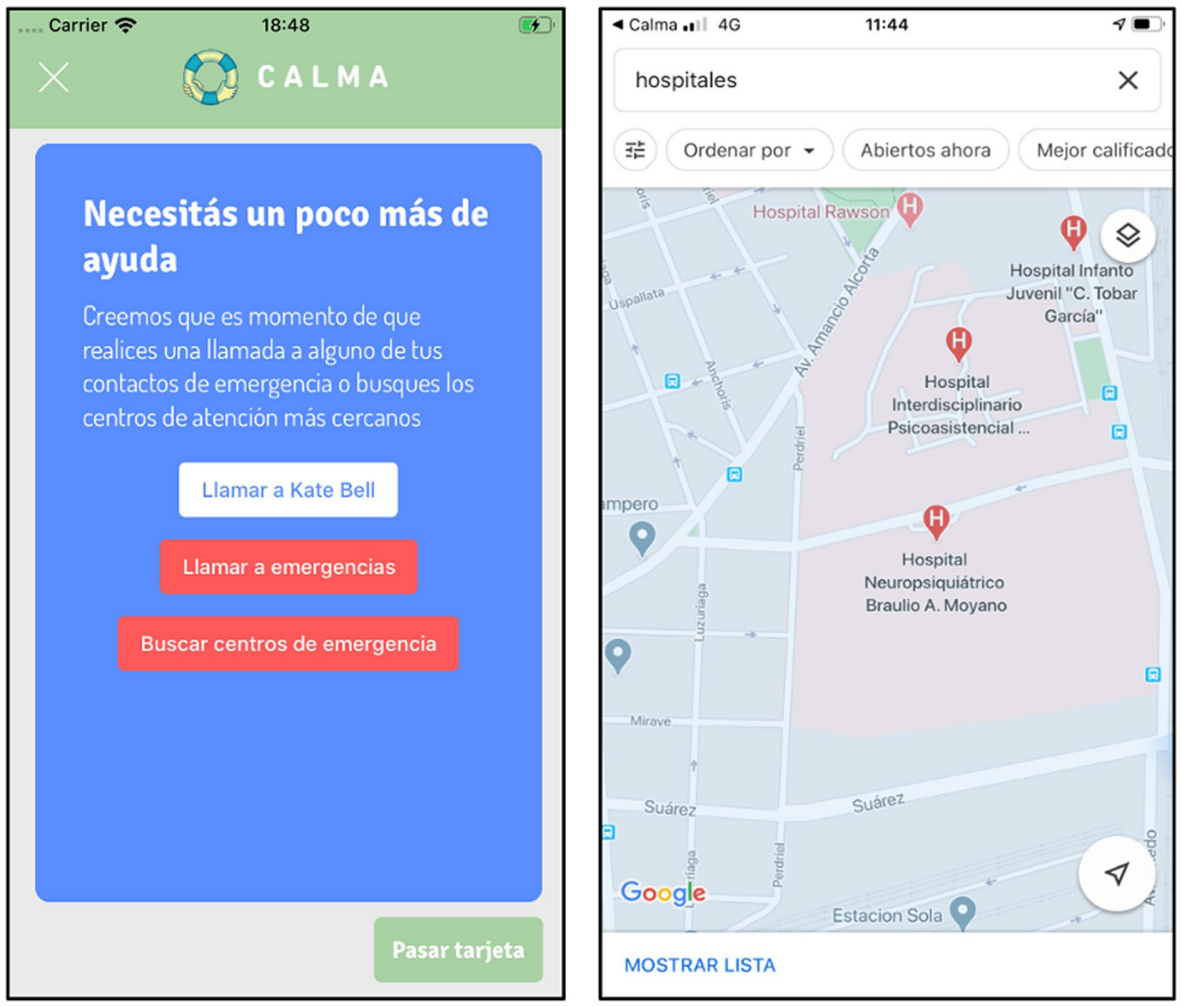
“I need help” mode of CALMA: Emergency Card.

“Emergency Card”: if distress worsens or does not diminish after three attempts, CALMA activates the “Emergency Card” (Figure 5). This card offers the user the option to make one or several calls emergency contacts previously loaded or standardized emergency numbers (e.g., 911). This card also provides the user the option to use the geolocation function, which uses Google Maps to show all emergency services near the user’s location so that he/she can consult personally.

The CALMA version used for this trial is identical to the one available for download.

This app was initially developed to be used by a mental health clinician as a complement to psychological and psychopharmacological treatments, but it can also be used by individuals who do not have access to the health system or who are afraid of being stigmatized, since it does not require training for its use and all the information is contained in the same app. The intervention was based on DBT’s biosocial theory and on the principles of learning that posits that the patient lacks skills to manage painful emotions. CALMA offers the possibility of putting these emotional regulation skills into practice, with instructions on how to carry them out, increasing the probability of incorporating these behaviors into the user’s behavioral repertoire through validation and/or reinforcement phrases as appropriate. In addition, before proposing skills, CALMA promotes the use of problem-solving strategies in each of the crises. The increase in perceived self-efficacy in crisis resolution and the learning of DBT skills can help the patient become less involved in behaviors related to suicide, through the increase in the ability to regulate emotionally as a mediator of the effect. CALMA also works between crisis situations by promoting activities aimed at reducing the subject’s vulnerability to emotional crises and providing psychoeducational content about suicide and its prevention.

The intervention includes different behavior change techniques such as problem solving (1.2.), reducing negative emotions (11.2.), distraction (12.4.), shaping knowledge (i.e., instruction on how to perform the behavior) (4.1.), non-specific reward (10.3.) and social support (unspecified) (3.1) from the BCT Taxonomy v1 (67).

Finally, CALMA complies with the current system and privacy recommendations for the design of mobile applications for suicide prevention (49).

### Outcomes measures

#### Primary outcome measure

Suicidal behavior will be assessed using the Spanish version of the Self-Injurious Thoughts and Behaviors Interview (SITBI) (68, 69). This is a structured interview consisting of 169 items divided into 5 modules that examine the presence, frequency and characteristics of 5 types of self-injurious behavior: (a) suicidal ideation; (b) suicide plans; (c) suicidal gestures; (d) suicide attempts and (e) self-harm. The SITBI conceptualizes suicide risk on a continuum, starting with suicidal ideation (“thoughts of killing yourself?”), possibly accompanied by a suicide plan [“think about how you might kill yourself (e.g., taking pills, shooting yourself) or work out a plan of how to kill yourself?”] and in some cases by suicide attempt [“made a suicide attempt (i.e., purposefully hurt yourself with at least some intent to die)?”]. The construction of the questions in the original SITBI is consistent with the commonly accepted definitions of each type of behavior. The administration of the scale takes between 3 and 15 min.

#### Secondary outcome measures

Secondary outcomes comprise the following:

##### Emotional dysregulation

Emotional dysregulation will be quantified with the validation in Spanish of the Emotional Regulation Difficulties Scale (DERS) (70). The original scale has 36 items with a Likert response format of 5 positions (from 1 = almost never, to 5 = almost always) and a structure of six factors: (1) Impulse control difficulties (6 items: “When I’m upset, I lose control over my behavior,” for example); (2) Limited access to emotion regulation strategies (8 items: “When I’m upset, I believe there is nothing I can do to make myself feel better,” for example); (3) Nonacceptance of emotional responses (6 items: “When I’m upset, I feel ashamed at myself for feeling that way,” for example); (4) Difficulty engaging in Goal-directed behavior (5 items: “When I’m upset, I have difficulty concentrating,” for example); (5) Lack of emotional awareness (6 reverse-scored items: “I am attentive to my feelings,” for example) and (6) Lack of emotional clarity (5 items: “I am confused about how I feel,” for example). The DERS has previously been used in pre-post intervention studies as a state variable of emotional dysregulation to assess effectiveness (71–74). This variable will be considered and analyzed as a quantitative variable.

##### Engagement with the app

Engagement will be determined by three parameters: acceptability, subjective use, and objective use. For acceptability, the User Experience Questionnaire will be used in its short version (UEQ-s) (75). The UEQ-s scale contains 8 items that evaluate the app’s pragmatic qualities (attractiveness, clarity and reliability) and hedonic qualities (novel, stimulating). Each item is scored on a Likert scale from 0 to 7, with antagonistic qualities being found at their extremes (0 and 7). Subjective use will be assessed with 5 directed questions are asked with the possibility of a dichotomous response (Yes/No); (“Did you use the app when you were in crisis?”; “In case you used it, was it useful for crisis management?”; “Did you use the app outside of times of crisis? crisis?”; “Do you consider that this modality “out of crisis” helped you to prevent the appearance of new crises?”; “Would you recommend the application to other people?”).

Objective use of the app will be obtained and recorded *via* the participant’s phone number. Specifically, use will be recorded when at least one of the following features of CALMA is used during the study period: opened a “Tips,” arrived at the “Emergency Card,” finished a crisis (due to success or abandonment), opened a notification, added a contact, reviewed their “Moments,” added a picture or content to their “Moments,” closed the app while it was in crisis and ended a crisis successfully. In addition, patients will be defined as “users” of the app if they met the following criteria: subjectively reported using the app in the survey and used at least one feature recorded by our objective measures. Finally, the relationship between the content of the app used by the participants and the primary outcome will be evaluated.

##### Time to psychiatric admission

At follow-ups and at the final interview, participants will be asked if they have had a psychiatric hospitalization for a self-injurious behavior during the follow-up period. This measure will be used to compare time to psychiatric admission in those participants who received the intervention with those participants who did not receive the app.

##### Sociodemographic variables

Sociodemographic variables are: age, sex, gender, sexual orientation, education level and treatment modality (outpatient/inpatient).

### Participant timeline

Clinicians will be asked to draw up a list of patients who have presented self-injurious behavior in the last month. With this list, the patients will be identified on the days that they attend the control by their psychiatrist or psychologist of each Service. After the usual control of the patient, one of the members of the research group will explain the purpose of the study to the participant and legal guardian. Next, the researcher will give them a copy of the consent and/or assent to read carefully and invite them to participate in the study. After the invitation, those who agree to participate will be scheduled for a 30-min interview with one of the research group members.

The first interview will be face-to-face and it will determine if the participant meets the inclusion criteria and sign the informed consent. Then, a series of clinical and demographic data will be requested and the Spanish version of the Self-Injurious Thoughts and Behaviors Interview (SITBI) and the Difficulties in Emotion Regulation Scale (DERS) will be administered. At the end of the interview, the follow up interviews will be scheduled and those patients randomized to the intervention group will have the application downloaded to their cell phones. In addition, they will be given a brief 5 to 10-min tutorial on how the app works, they will be given written material on its functioning, and they will be directed to the website where there will be explanatory material.^1^

Follow-up interviews (day-30 and day-60): those will be conducted *via* video call according to the epidemiological context of the COVID-19 pandemic. During these calls, only the SITBI will be administered and participants will be asked if they have had any hospitalization due to suicidal behavior in that period, and if so, the date of admission. In the intervention group, to guarantee engagement with the CALMA app, the rater will explore whether or not the participant has used the application. In case of not using it, the obstacles and the alternatives to overcome them will be determined through a brief protocol (DBT missing links strategy) to reinforce the use of the application during the next evaluation period. At the same time, the technical team will work throughout the study to guarantee the proper functioning of the app.

**Figure 6:**
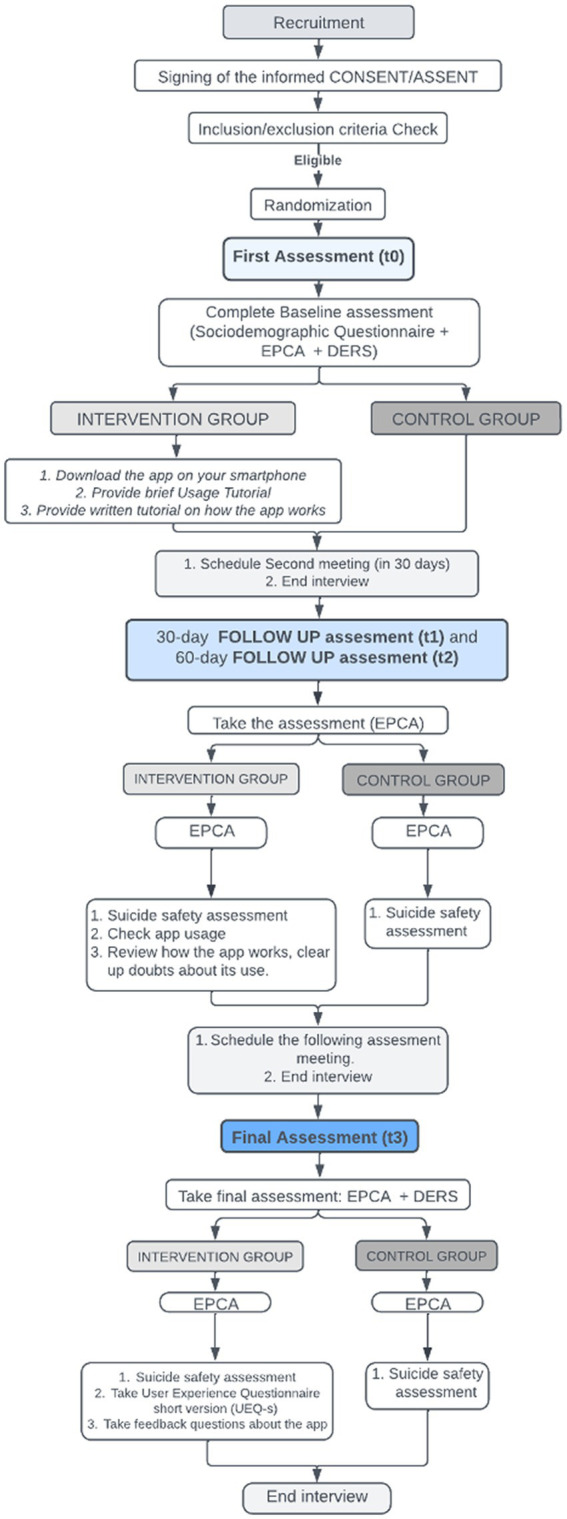
Flow of participants.

The final interview (day-90) will be face-to-face or by video call depending on the epidemiological context of the COVID-19 pandemic. The SITBI, the DERS and, to those who received the app, the User Experience Questionnaire short version (UEQ-s) and a series of questions related to the opinion about the app will be administered. At the end of this interview, we will obtain data from the patient about the frequency of use of the app (subjective use report) and our information technology team will also obtain data on the use of the different functionalities of the app from each participant (objective use report). A flowchart describing how an individual participant will progress through the phases of the research process is outlined in Figure 6.

### Sample size

The sample size was calculated using the G*Power 3.1.9.4 statistical software according to the main objective. Considering a confidence level of 95%, a power of 80% and an effect size *d* = 0.85, 25 individuals per group are needed to detect a significant difference in the median frequency of self-injurious behaviors with the two tailed Mann–Whitney U test. Assuming a proportion of lost in the follow-up of 15%, then the study would have to start with 29 individuals per group.

### Recruitment

A non-probabilistic sampling procedure will be applied, where outpatients or hospitalized patients of the Public Mental Health Services who have presented some type of self-injurious behavior (with or without suicidal intent) the month prior to the evaluation will be invited to participate.

Therefore, the study will use a primary data source. For this, one of the psychiatrists of the research group will explain the purpose of the study and will invite each of the participants and their legal guardians to participate in the study. It will be made clear that regardless of the arm of the study to which each subject is assigned, at the end of the study all participants will be able to access the application free of charge. After the invitation, those who agree to participate will be interviewed by one of the members of the research group.

### Randomization

To perform the simple randomization of the treatments, the RStudio software version 1.4.1106 was used. For each of the hospitals, a patient ID vector was created (45 rows). Then, the ID of each hospital was randomly assigned a treatment, generated from a Bernoulli distribution with *p* = 0.5. Previously, a starting seed was set to ensure the reproducibility of the algorithm. The lists obtained were exported to a spreadsheet in Microsoft Excel. Each responsible investigator in each hospital received the list of the randomization sequence by email from the study coordinator (DER).

### Data management

All research data collected in this trial will be stored using a unique participant ID code, and will be stored in numerical order and stored in a secure and accessible place and manner in the Institute of Pharmacology od the School of Medicine of the University of Buenos Aires. Participant files will be maintained in storage for a period of 3 years after completion of the study.

The data manager will be responsible for uploading the collected data to a secure database, and will be responsible for securely transferring data to the research team. Data will be exported into appropriate statistical software for analysis. Only authorized study team members are able to view data. If there a rationale (e.g., participant request, unexpected adverse events, aggregate data indicates self-harm above the rates expected from this group) the research team will make the decision to terminate the trial if necessary. All adverse events attributable to a study intervention will be recorded and reported to the ethics committee of each institution. Participants may be withdrawn from the study if they experience a serious adverse event that is attributable to a study intervention or procedure, or means they can no longer participate in the study. Participants may also self-withdraw consent to take part. Participants that are withdrawn from the study will not be replaced.

### Statistical analysis

To describe the sociodemographic characteristics of the patients in the two groups, absolute and relative frequencies (%) will be used for the qualitative variables, and the mean and standard deviation will be used for the only quantitative variable (age) if it presents a normal distribution according to the Shapiro–Wilk test. In case of presenting a non-normal distribution, the median and the interquartile range will be computed.

To compare the sociodemographic characteristics between the groups, the chi-square or Fisher’s exact test will be used for the qualitative variables, and the student’s t-test or the Mann–Whitney U test will be computed for the quantitative variable.

For all patients and at each follow-up moments, the difference between the frequency of each suicidal behavior and the baseline frequency will be calculated. Then it will be evaluated if these differences follow a normal distribution (Shapiro–Wilk test). In this case, the mean differences for each behavior between both groups will be compared using the t-Student test. Otherwise, the medians will be compared with the Mann–Whitney U test. The *p*-values obtained will be corrected according to the Bonferroni criterion.

To assess whether the change in emotional regulation mediates the change in the frequencies of each suicidal behavior, the difference between the value of the DERS scale at the last follow-up and the baseline will be calculated, and the Sobel test will be used on a regression model whose dependent variable will be the difference in the frequency of each behavior and the independent variable will be the treatment. Previously, the probability distribution of the quantitative variables will be evaluated and it will be considered whether it is necessary to carry out any transformation of data to guarantee compliance with the assumptions of the regression model.

Both for the frequencies of the different suicidal behaviors and for the DERS scale, the missing values will be imputed according with the multiple imputation method using chained equations (MICE) with appropriate models for each variable to be imputed, considering sociodemographic characteristics, the hospital of recruitment and the baseline measurements of frequencies and DERS scale as independent variables.

Regarding acceptability, the UEQ Data Analysis Tool offers a benchmark to classify the application in 5 categories of 6 scales: excellent, good, above average, below average and bad allowing conclusions to be drawn about the relative quality of CALMA compared to other products.^2^

To evaluate and compare the time to readmission in patients enrolled during hospitalization, a Cox regression model will be adjusted, considering the treatment of each patient as the independent variable. For this, the time elapsed from study entry to readmission (event) or to loss of follow-up or final of the study (censoring) will be calculated. In all cases, a value of *p* less than 0.05 will be considered significant. Statistical analysis will be performed with RStudio 1.4.1106.

### Safety procedure

A strict suicide safety protocol will be followed given our participant eligibility criteria. Patient safety will be the highest priority in this study. Included in the CALMA app’s “Emergency Card” is a linked directory of major crisis helplines and a geolocation feature of emergency services near the user’s location, allowing participants to connect directly to these services.

A suicide safety assessment will be carried out in each interview, where the SITBI will be administered. Under the circumstance that the patient is at risk, the interviewer (a psychiatric member of the research group) will alert the parent and indicate that he/she accompany the participant to the Emergency Department of the center where the recruitment was carried out to be evaluated by a mental health professional. The interviewer also will alert the study coordinator (DER) and will alert the research collaborator at each recruitment center. Each collaborator will immediately notify to the emergency room physician on call that day for an acute psychiatric assessment so that said clinician can contact the participant’s parent, guaranteeing the presence of the patient in the emergency department. In addition, each collaborator will notify the patient’s pediatric medical team of each hospital.

### Patient and public involvement

In the previous CALMA pilot study carried out by this research group, feedback was collected with the experiences and preferences of the participants in the intervention group, which have been taken into account for the current version of the app. Patients were not involved in other aspects of the design, or conduct, or reporting, or dissemination plans of our research. In case the current study shows positive results, the app will be downloaded to the smartphones of the control group participants for use after follow-up.

## Discussion

Smartphones provide promising opportunities for wider dissemination of and access to suicide prevention interventions by improving help-seeking behavior and providing low-cost real-time help (48). There are backgrounds of interactive apps with DBT tools, such as DBT Coach, but designed specifically to augment skills generalization through interactive coaching in DBT skills as an adjunct to standard DBT. Particularly, use of the DBT Coach was not related to any treatment outcomes, except for reductions in NSSI (76). Others apps have incorporated behavioral interventions such as a diary as an adjunct to DBT treatment (77). In the same line, Schiffler et al. have shown the implementation of a Mobile DBT App for young adults (aged 18 to 23 years) with a focus on suicidality and NSSI, in a natural setting, without showing effectiveness results (78).

A recent systematic review measured the effectiveness of mobile apps for Monitoring and Management of Suicide Crisis (79). Of the 32 included studies, 16 evaluated feasibility and acceptability of those mobile apps, 10 studies assessed the effectiveness of mobile app in preventing suicide and six studies described randomized control trial protocols not yet implemented. Most of the studies took place in Europe (*n* = 13) and the United States (*n* = 9); only two of them were about apps in Spanish. First, MEmind, a tracking non-intervention mobile app with EMA (Ecological Momentary Assessment), and CALMA. Of the 16 studies that evaluated feasibility and acceptability, four included adolescents with suicidal ideation or behaviors, some of which included DBT skills within their features or personal problem-solving strategies. Two of them also included adults, and only the other two focused specifically on adolescents. Of the 10 efficacy studies, two included adolescents who attempted suicide. The first was a one group pre–post test pilot study with only 3 participants. The second one, an RCT of 66 hospitalized suicidal adolescents that reported no significant differences in suicidal outcomes. It is worth clarifying that the intervention was a face-to-face plus a mobile app after discharge intervention.

In a prior pilot study, the CALMA app has shown initial evidence of safety and acceptability of the app for reducing self-injurious thoughts and behaviors when used as an adjunct to conventional DBT therapy within 4 weeks of follow-up (52). This study has shown good acceptability of CALMA as an adjunct to therapy targeting suicidal and non-suicidal self-injured behaviors. Furthermore, a high probability of decreased in SI, suicidal plan, suicidal gesture, thoughts about Non-Suicidal Self-Injury (NSSI) and NSSI pre-and post-intervention was observed in the group that received CALMA and a high probability of a greater decrease in SI and suicidal gesture before and after the intervention for the group that received CALMA compared to the comparison group.

This protocol describes a parallel-arm randomized controlled trial, which aims to test the efficacy, safety and acceptability of the CALMA application as an adjunct to therapy to reduce suicidal and non-suicidal self-injurious behaviors in adolescents.

This research will fill important gaps about the engagement and effectiveness of a DBT skills-based application in adolescents. Firstly, there are few studies of apps that focus on adolescents with suicidal behaviors, a population at higher risk of suicide and with distinctive characteristics (80); only some have the characteristic of being interactive with the user. Second, of the few studies that did measure effectiveness in this population, the results were negative. Third, this study is particularly relevant to young people given their widespread use of mobile technology, while there are currently no available mobile applications in Spanish at all that attempt to reduce suicidal behavior in this population (35, 81). Fourth, having a tool that can help reduce the probability of hospitalization for suicidal behavior that is economical, universal, and that is at the service of the crisis, such as an application, would undoubtedly democratize access to specific and effective intervention. Fifth, if the exploratory objective of this study demonstrates that the reduction of self-injurious behaviors is mediated by the improvement of emotional regulation, an app such as CALMA can be an invaluable tool to increase the capacity (skills) to manage intense and painful emotions.

The potential benefits for patients who have the app are that they will have a helpful tool to resolve self-injurious crises. In addition, if the study demonstrates the app’s effectiveness, the results may have potential benefits from a social point of view, since the app will be free to use.

In addition, this evidence will be important to add an innovative and alternative tool to the treatment of suicidal behavior in this population in the public health sector, especially from low and middle-income countries in Latin America, since no study at all was carried out in this region of the world.

Smartphone app-based self-guided psychological strategies for suicidal thoughts and behaviors have the potential to increase access to treatment and reduce barriers to help-seeking by providing accessible, anonymous and timely support, which could then reduce suicide risk, especially in scenarios like the COVID-19 pandemic (47, 82, 83).

## Ethics and dissemination

The present protocol and applied informed consent forms were approved concerning their content and compliance with ethical regulations by the Elizalde Hospital Ethical Committee and the Institutional Committee for the Review of Research Protocols. The study is carried out with principles enunciated in the Declaration of Helsinki. Furthermore, the study will be carried out following the “Law on the Protection of the Rights of Subjects in Health Research” (Law 3,301) and the “Law on the Protection of Personal Data” (Law 25.326) of Argentina. In addition, all rights protection guidelines for human volunteers will be followed.

All researchers participating in the project are trained in research ethics and are familiar with current regulations. Only authorized study personnel will have access to protect the confidentiality of the data in the electronic database where the data is found. The information that will be obtained in the database will not have personal identification; each study participant will be assigned a unique identification code (ID).

The inclusion criteria for the study imply that the patients included in it must have presented some self-injurious behavior the month before the evaluation; therefore, they are high-risk patients. For this reason, they will be receiving treatment by the Mental Health team during the study period. The additional risks for the intervention (the use of the CALMA app) are low for this study, in previous studies we have shown that the app does not increase the risk of suicide.

The results of the planned analyzes will be published in a peer-reviewed journal.

## Ethics statement

The Elizalde Hospital Ethical Committee (ID 884; Effectiveness, safety and acceptability of a mobile phone application for the reduction of self-injurious thoughts and behaviors in adolescents: a randomized controlled study) and the Institutional Committee for the Review of Research Protocols (CIRPI; ID 2/2020 Effectiveness, safety and acceptability of a mobile phone application for the reduction of self-injurious thoughts and behaviors in adolescents: a randomized controlled study) have approved this study. The participants and legal guardian will provide written informed consent to participate in this study.

## Author contributions

All authors listed have made a substantial, direct, and intellectual contribution to the work and approved it for publication.

## Funding

This work was supported by “Salud Investiga” Call 2021–2022–Ministry of Health of the Argentine Nation, grant number NRU 409, under the name: “Effectiveness, safety and acceptability of an application for mobile phones to reduce self-injurious thoughts and behaviors in adolescents: a randomized controlled study”–proyecto de investigación clínica de implementación financiado por la Secretaria de Gobierno de Salud. This funding source had no role in the design of this study and will not have any role during its execution, analyzes, interpretation of the data, or decision to submit results.

## Conflict of interest

FD, DR, and RF are the owners of CALMA license.

The remaining authors declare that the research was conducted in the absence of any commercial or financial relationships that could be construed as a potential conflict of interest.

## Publisher’s note

All claims expressed in this article are solely those of the authors and do not necessarily represent those of their affiliated organizations, or those of the publisher, the editors and the reviewers. Any product that may be evaluated in this article, or claim that may be made by its manufacturer, is not guaranteed or endorsed by the publisher.

## Footnotes

^1^ www.appcalma.com

^2^ http://www.ueq-online.org/
